# Fatty acid composition of lipids in pot marigold (*Calendula officinalis* L.) seed genotypes

**DOI:** 10.1186/1752-153X-7-8

**Published:** 2013-01-17

**Authors:** Francisc V Dulf, Doru Pamfil, Adriana D Baciu, Adela Pintea

**Affiliations:** 1 University of Agricultural Sciences and Veterinary Medicine, Manastur 3-5, Cluj-Napoca, 400372, Romania

**Keywords:** *Calendula officinalis* L., Conjugated linolenic acids, Pot marigold, Seed oils, Fatty acids, Polar lipids, Triacylglycerols, Sterol esters, GC-MS

## Abstract

**Background:**

*Calendula officinalis* L. (pot marigold) is an annual aromatic herb with yellow or golden-orange flowers, native to the Mediterranean climate areas. Their seeds contain significant amounts of oil (around 20%), of which about 60% is calendic acid. For these reasons, in Europe concentrated research efforts have been directed towards the development of pot marigold as an oilseed crop for industrial purposes.

**Results:**

The oil content and fatty acid composition of major lipid fractions in seeds from eleven genotypes of pot marigold (*Calendula officinalis* L.) were determined. The lipid content of seeds varied between 13.6 and 21.7 g oil/100 g seeds. The calendic and linoleic acids were the two dominant fatty acids in total lipid (51.4 to 57.6% and 28.5 to 31.9%) and triacylglycerol (45.7 to 54.7% and 22.6 to 29.2%) fractions. Polar lipids were also characterised by higher unsaturation ratios (with the PUFAs content between 60.4 and 66.4%), while saturates (consisted mainly of palmitic and very long-chain saturated fatty acids) were found in higher amounts in sterol esters (ranging between 49.3 and 55.7% of total fatty acids).

**Conclusions:**

All the pot marigold seed oils investigated contain high levels of calendic acid (more than 50% of total fatty acids), making them favorable for industrial use. The compositional differences between the genotypes should be considered when breeding and exploiting the pot marigold seeds for nutraceutical and pharmacological purposes.

## Background

*Calendula officinalis* L. (pot marigold), a member of the *Asteraceae* family, is an annual aromatic herb with yellow or golden-orange flowers, native to the Mediterranean climate areas, being also successfully cultivated in temperate regions of the Earth for ornamental and medicinal purposes [1]. The species have been reported to contain a variety of phytochemicals, including carbohydrates, lipids, phenolic compounds, steroids, terpenoids, tocopherols, carotenoids and quinones [2-5] with potential health benefits [1,6-10].

Besides the usual fatty acids, a few plants are capable to biosynthesize some unusual fatty acids, with special chemical structure. Usually these fatty acids accumulate in storage tissues, while in green organs they are absent or present in very small amounts. The presence of unusual fatty acids is genetically determined and they are highly significant indicators of phylogenetic relationships [11,12]. The seeds of pot marigold have a significant oil content (around 20%), of which about 60% is the unusual calendic acid (8 *t*, 10 t, 12c-18:3) [13-16]. Several studies demonstrated that calendic acid is synthesized in *Calendula* seeds via desaturation of linoleic acid [17-21]. Due to its special structure – with three conjugated double bonds – calendic acid and *Calendula* seeds oil exhibit interesting chemical and physiological properties.

The seed oils such of *Calendula officinalis* L., *Momordica charantia* L. or *Aleurites fordii* Hemsl., rich in conjugated linolenic acids (CLNAs) have a high rate of oxidation and are used as raw materials in paints and coatings industry, and have applications in the manufacture of cosmetics and some industrial polymers [19,22-24]. For these reasons, in the last few years, a concentrated research effort in Europe has been directed towards the development of *Calendula officinalis* L. as an oilseed crop for industrial purposes [25] and for the engineering of transgenic plants containing the metabolic route for the conjugated fatty acids biosynthesis [26,27].

The increasing interest for plants producing conjugated fatty acids is also motivated by the recent findings related to their biological effects. It has been shown that CLNAs have an important body fat-lowering effect [28] and possess anti-carcinogenic properties, exhibiting apoptotic activity against a wide variety of tumor cells, such as the U-937 human leukemic cancer cell line and the colon cancer cells (Caco-2) [24,29,30]. Bhaskar et al. [31] observed that the *trans* CLNAs exhibited stronger growth inhibition and more DNA fragmentation in human colon cancer cells than corresponding *cis* CLNA isomers.

To our knowledge, all the studies, excepting two short reports of Ul’chenko et al. [32] and Pintea et al. [33], respectively, conducted on marigold seed oils determined the fatty acid contents by analyzing only the total lipid matrix.

Therefore, the aim of the present investigation was to compare the oil content and fatty acid compositions of total lipids (TLs), triacylglycerols (TAGs), polar lipids (PLs) and sterol esters (SEs) in seeds of eleven pot marigold genotypes from six different locations in Europe, grown in the Transylvanian region (Romania). The information obtained is helpful to identify suitable genotypes for use in breeding programs of *Calendula officinalis*.

## Results and discussion

### Oil contents

**Figure 1:**
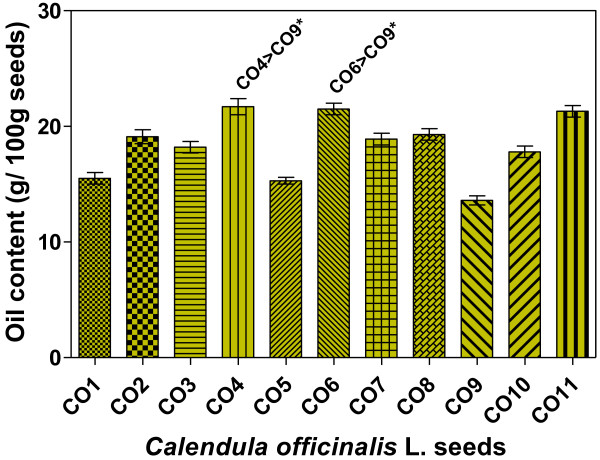
**The oil content of Pot marigold (*Calendula officinalis *L.) seeds.** CO1- CO11, pot marigold (*Calendula officinalis* L.) genotypes. Results are given as mean ± SD (n = 3); * - significant difference, *p* < 0.05 (using "Kruskal-Wallis non-parametric test" followed by "Dunn's Multiple Comparison Test").

The oil (total lipids) contents in eleven genotypes of pot marigold (*Calendula officinalis* L.) (CO) seeds are presented in Figure 1.The values were found to vary between 13.6- 21.7 (g oil/100 g seeds). There were no significant differences (*p* < 0.05) among genotypes, except for oil contents of samples CO4 and CO6 versus CO9. The highest amounts of oils were found in the CO4 (21.7 g/100 g), CO6 (21.5 g/100 g) and CO11 (21.3 g/100 g), whereas the genotypes CO1 (15.5 g/100 g), CO5 (15.3 g/100 g) and CO9 (13.6 g/100 g), exhibited the lowest contents of the TLs. These values were similar to those reported by Cromack and Smith [25] but much higher than those observed by Ozgul- Yucel (5.9% oil in Turkish *Calendula* seeds) [34] and Angelini et al. (5.4% oil in Italian CO seed crops from 1994) [35]. The TLs content of the analyzed CO seeds in this study were also comparable with those of some non-conventional vegetable oil sources with unique phytochemical compositions, such as bitter gourd (21%), cherry laurel (18.3%), pomegranate (18.1%), blackthorn (16.5%), linseed dodder (15.5-20.7%), and coriander (12.7-18%) seeds [34,35].

### Fatty acid composition

The total lipid fatty acid composition as well as the fatty acid composition of TAGs, PLs and SEs of the analyzed pot marigold seed oils is presented in Tables 1 and 2.

**Table 1:** Fatty acid composition (%) in total lipids and individual lipid classes of different genotypes of pot marigold seed oils

**Species**	**Fatty acids (%w/w of total fatty acids)**																		
	**12:0**	**14:0**	**15:0**	**16:1 (n-9)**	**16:1 (n-7)**	**16:0**	**17:0**	**18:2 (n-6)**	**18:1 (n-9)**	**18:1 (9** ***t*) (n-9)**	**18:2 (9** ***t*,12** ***t*) (n-6)**	**18:0**	**18:3 (n-3)**	**18:3 (8** ***t*,10** ***t*,12*c*) (n-6)**	**20:1 (n-9)**	**18:3 (8** ***t*,10** ***t*,12** ***t*), (n-6)**	**20:0**	**9-HODE**	**22:0**
**CO1**																			
TL	0.02	0.18	0.01	0.03	0.08	4.05	0.03	30.05	4.99	0.51	tr	1.87	0.09	55.93	0.14	0.85	0.36	0.63	0.18
TAG	nd	0.15	0.08	0.03	0.08	5.62	tr	29.18	5.76	0.50	nd	2.62	0.15	54.67	0.10	0.60	0.46	nd	nd
PL	0.64	3.88	0.27	0.31	0.15	17.08	nd	60.78	6.49	0.70	tr	3.72	nd	4.29	tr	0.43	0.59	tr	0.67
SE	0.62	2.86	nd	tr	nd	20.02	nd	34.91	11.02	0.12	nd	2.53	nd	4.64	nd	nd	2.45	nd	20.83
**CO2**																			
TL	0.03	0.30	0.01	0.03	0.07	4.33	0.04	28.50	4.49	0.53	0.02	1.84	0.10	57.22	0.16	0.77	0.42	0.89	0.25
TAG	nd	0.13	0.02	0.04	0.18	7.98	0.04	25.10	9.10	1.10	nd	3.95	0.09	50.03	0.47	0.70	1.07	nd	nd
PL	0.41	4.44	0.15	0.16	0.08	20.61	0.17	55.02	7.04	1.01	1.06	3.94	nd	3.98	0.07	0.40	0.61	0.17	0.68
SE	0.91	3.05	nd	0.39	nd	20.23	nd	33.75	10.32	0.14	nd	2.97	nd	4.94	nd	nd	2.53	nd	20.77
**CO3**																			
TL	0.01	0.15	0.01	0.03	0.07	3.93	0.02	31.79	5.06	0.54	0.06	1.73	0.10	54.07	0.15	0.79	0.38	0.95	0.17
TAG	nd	0.14	0.02	0.04	0.18	10.21	0.07	24.30	10.02	0.73	nd	4.08	0.10	48.53	0.45	0.53	0.60	nd	nd
PL	0.17	2.45	0.12	0.13	0.12	17.66	0.15	61.33	6.88	1.13	0.76	3.43	nd	3.86	tr	0.46	0.50	0.13	0.72
SE	0.72	2.53	nd	0.33	nd	22.77	nd	32.34	12.00	0.20	nd	2.93	nd	2.69	nd	nd	2.49	nd	21.00
**CO4**																			
TL	0.02	0.19	0.01	0.04	0.06	4.55	0.02	28.52	4.44	0.41	0.01	1.77	0.13	57.63	0.14	0.91	0.35	0.66	0.12
TAG	nd	0.15	0.03	0.08	0.18	8.59	0.04	23.32	10.79	1.20	nd	4.20	0.08	49.02	0.53	0.51	1.28	nd	nd
PL	0.22	2.29	0.12	0.26	0.12	16.55	0.16	61.15	8.13	1.30	0.74	3.18	nd	4.19	0.10	0.34	0.46	0.16	0.53
SE	1.24	2.05	0.38	0.78	nd	21.78	nd	32.41	11.80	1.53	nd	3.51	nd	3.93	nd	nd	2.68	nd	17.91
**CO5**																			
TL	0.02	0.22	0.01	0.03	0.07	4.22	0.03	31.47	6.19	0.55	0.07	1.88	0.11	52.52	0.15	0.83	0.40	0.98	0.25
TAG	nd	0.15	0.02	0.05	0.25	8.15	0.04	24.75	13.27	1.15	nd	4.24	0.07	46.00	0.58	0.38	0.90	nd	nd
PL	0.26	3.23	0.16	0.17	0.12	20.31	0.19	57.96	7.69	1.20	0.77	3.68	nd	2.66	0.04	0.16	0.59	0.11	0.70
SE	1.01	2.11	nd	tr	nd	19.70	nd	33.52	10.31	0.37	nd	4.65	nd	4.60	nd	nd	2.80	nd	20.93
**CO6**																			
TL	0.02	0.21	0.02	0.04	0.06	4.48	0.04	29.49	5.26	0.55	0.02	1.83	0.09	55.80	0.13	0.66	0.34	0.89	0.07
TAG	nd	0.16	0.02	0.07	0.22	9.55	0.06	22.60	12.01	1.34	nd	4.42	0.06	47.85	0.48	0.33	0.83	nd	nd
PL	0.35	2.83	0.21	0.18	0.10	20.14	0.21	59.04	7.51	1.24	0.61	3.72	nd	2.61	tr	0.13	0.59	0.06	0.47
SE	1.59	2.90	nd	tr	nd	20.37	nd	32.33	12.29	0.17	nd	3.97	nd	3.75	nd	nd	2.85	nd	19.78
**CO7**																			
TL	0.02	0.33	0.02	0.03	0.08	4.54	0.04	30.26	6.04	0.57	1.66	1.84	0.08	53.17	tr	0.46	0.38	0.38	0.11
TAG	nd	0.16	0.02	0.10	0.22	8.62	0.08	24.62	12.65	1.13	nd	4.03	0.08	46.46	0.57	0.44	0.82	nd	nd
PL	0.29	2.74	0.13	0.16	0.14	19.90	0.22	60.46	7.20	1.13	tr	3.33	nd	2.77	0.11	0.13	0.53	0.11	0.65
SE	2.19	2.80	nd	0.71	nd	18.31	nd	33.89	10.83	0.22	nd	3.30	nd	3.77	nd	nd	3.32	nd	20.66
**CO8**																			
TL	0.05	0.39	0.01	0.02	0.11	4.11	0.03	31.86	6.25	0.49	1.80	1.99	0.11	51.47	0.02	0.48	0.41	0.24	0.17
TAG	nd	0.16	0.02	0.05	0.29	8.26	0.05	23.37	14.32	1.11	nd	4.77	0.06	45.73	0.53	0.43	0.85	nd	nd
PL	0.28	2.34	0.16	0.25	0.14	19.37	0.22	58.97	8.82	1.22	0.24	3.63	nd	2.91	tr	0.19	0.54	0.13	0.59
SE	1.75	3.14	nd	1.51	nd	23.77	nd	27.46	10.07	0.48	nd	3.03	nd	4.74	nd	nd	3.76	nd	20.29
**CO9**																			
TL	0.03	0.19	0.02	0.02	0.10	3.98	0.05	30.81	4.98	0.53	1.34	1.87	0.12	54.21	tr	0.58	0.39	0.27	0.51
TAG	nd	0.11	0.03	0.04	0.25	6.49	0.07	27.68	8.94	1.01	nd	3.59	0.10	50.06	0.47	0.53	0.63	nd	nd
PL	0.19	1.14	0.16	0.26	0.23	20.82	0.24	59.34	7.28	1.22	0.88	3.58	nd	2.95	0.05	0.17	0.62	0.10	0.77
SE	1.50	3.58	nd	1.05	nd	20.42	nd	32.38	9.68	0.24	nd	2.88	nd	3.69	nd	nd	3.37	nd	21.21
**CO10**																			
TL	0.02	0.16	0.02	0.02	0.12	3.86	0.03	30.99	4.97	0.53	1.71	1.87	0.11	53.88	tr	0.65	0.36	0.29	0.41
TAG	nd	0.08	0.01	0.05	0.31	6.99	0.05	27.93	9.09	1.06	nd	3.76	0.07	49.18	0.43	0.41	0.58	nd	nd
PL	0.12	0.95	0.15	0.25	0.25	20.28	0.27	61.51	6.98	1.16	0.35	3.51	nd	2.84	0.05	0.20	0.55	0.09	0.49
SE	1.86	3.31	nd	tr	nd	20.83	nd	32.16	10.11	0.23	nd	3.47	nd	3.43	tr	nd	3.62	nd	20.98
**CO11**																			
TL	0.03	0.38	0.02	0.04	0.05	4.43	0.04	30.32	4.78	0.56	0.62	1.75	0.11	55.32	0.05	0.61	0.39	0.21	0.31
TAG	nd	0.13	0.02	0.07	0.21	7.79	0.04	27.44	8.36	1.15	nd	3.43	0.09	49.85	0.39	0.48	0.55	nd	nd
PL	0.33	2.36	0.15	0.45	0.09	20.98	0.25	59.55	7.71	1.24	tr	3.31	nd	2.34	tr	0.13	0.51	0.04	0.56
SE	1.57	3.36	nd	tr	nd	20.93	nd	31.84	10.88	0.19	nd	3.26	nd	3.19	nd	nd	3.61	nd	21.17

The values represent the means of three samples, analyzed individually in triplicate (n = 3x3).

CO1- CO11, pot marigold (*Calendula officinalis* L.) genotypes.

TL- total lipids, TAG- triacylglycerols, PL- polar lipids, SE- sterol esters, nd- not detected, tr- trace.

**Table 2:** The composition (%) of fatty acid classes in total lipids and major lipid fractions from different genotypes of pot marigold seed oils

**Species**	**Fatty acids (%w/w of total fatty acids)**										
	**∑ SFAs**		**∑ MUFAs**		**∑ PUFAs**		**∑ VLCSFAs (≥20C)**		**∑CLNAs**		**PUFAs/SFAs**
	**Mean**	**SD**	**Mean**	**SD**	**Mean**	**SD**	**Mean**	**SD**	**Mean**	**SD**	
**CO1**											
TL	6.70_d_	0.25	5.75_d_	0.18	86.83_a_	2.25	0.54_b_	0.02	56.78_a_	1.76	12.96_a_
TAG	8.93_c_	0.35	6.47_c_	0.20	84.45_a_	2.30	0.46_b_	0.03	55.27_a_	1.55	9.46_b_
PL	26.85_b_	1.11	7.65_b_	0.22	65.50_b_	1.85	1.26_b_	0.04	4.72_b_	0.15	2.44_c_
SE	49.31_a_	1.68	11.14_a_	0.32	39.55_c_	1.68	23.28_a_	1.05	4.64_b_	0.12	0.80_d_
**CO2**											
TL	7.23_d_	0.22	5.27_c_	0.15	86.52_a_	2.30	0.67_b_	0.03	57.99_a_	1.65	11.97_a_
TAG	13.19_c_	0.30	10.89_a_	0.22	75.83_b_	2.27	1.07_b_	0.03	50.73_b_	1.38	5.75_b_
PL	31.01_b_	1.10	8.36_b_	0.18	60.46_c_	2.10	1.29_b_	0.05	4.38_c_	0.12	1.95_c_
SE	50.46_a_	1.80	10.85_a_	0.20	38.69_d_	1.50	23.30_a_	0.70	4.94_c_	0.15	0.77_d_
**CO3**											
TL	6.39_d_	0.19	5.85_d_	0.16	86.70_a_	2.38	0.54_b_	0.03	54.85_a_	1.32	13.56_a_
TAG	15.12_c_	0.35	11.42_b_	0.25	73.36_b_	1.95	0.60_b_	0.03	49.06_b_	1.75	4.85_b_
PL	25.20_b_	0.80	8.26_c_	0.18	66.41_c_	1.55	1.22_b_	0.04	4.32_c_	0.11	2.64_c_
SE	52.44_a_	1.60	12.53_a_	0.30	35.03_d_	1.15	23.49_a_	0.56	2.69_c_	0.10	0.67_d_
**CO4**											
TL	7.04_d_	0.16	5.09_d_	0.15	87.08_a_	2.42	0.47_b_	0.02	58.54_a_	1.58	12.37_a_
TAG	14.29_c_	0.31	12.78_b_	0.23	72.85_b_	2.10	1.28_b_	0.03	49.53_b_	1.65	5.10_b_
PL	23.51_b_	0.62	9.91_c_	0.22	66.42_c_	1.60	0.99_b_	0.03	4.53_c_	0.14	2.83_c_
SE	49.55_a_	1.55	14.11_a_	0.35	36.34_d_	1.11	20.59_a_	0.50	3.93_c_	0.11	0.73_d_
**CO5**											
TL	7.03_d_	0.17	6.99_d_	0.15	84.89_a_	2.30	0.66_b_	0.04	53.35_a_	1.25	12.08_a_
TAG	13.50_c_	0.28	15.30_a_	0.32	71.13_b_	2.00	0.90_b_	0.04	46.38_b_	1.60	5.27_b_
PL	29.12_b_	0.88	9.22_c_	0.20	61.55_c_	1.50	1.29_b_	0.03	2.82_c_	0.12	2.11_c_
SE	51.20_a_	1.65	10.68_b_	0.25	38.12_d_	1.20	23.73_a_	0.65	4.60_c_	0.14	0.74_d_
**CO6**											
TL	7.01_d_	0.20	6.03_d_	0.18	85.98_a_	2.35	0.41_b_	0.02	56.47_a_	1.35	12.26_a_
TAG	15.04_c_	0.32	14.12_a_	0.35	70.78_b_	2.10	0.83_b_	0.03	48.18_b_	1.62	4.71_b_
PL	28.52_b_	0.88	9.03_c_	0.25	62.39_c_	1.60	1.06_b_	0.02	2.74_c_	0.08	2.19_c_
SE	51.46_a_	1.70	12.46_b_	0.34	36.08_d_	1.15	22.63_a_	0.60	3.75_c_	0.09	0.70_d_
**CO7**											
TL	7.27_d_	0.22	6.72_d_	0.20	85.55_a_	2.40	0.48_b_	0.02	53.63_a_	1.30	11.77_a_
TAG	13.73_c_	0.30	14.67_a_	0.33	71.52_b_	2.15	0.82_b_	0.03	46.90_b_	1.25	5.21_b_
PL	27.79_b_	0.80	8.74_c_	0.24	63.36_c_	1.70	1.18_b_	0.03	2.90_c_	0.10	2.28_c_
SE	50.58_a_	1.48	11.76_b_	0.20	37.66_d_	1.10	23.98_a_	0.70	3.77_c_	0.15	0.74_d_
**CO8**											
TL	7.15_d_	0.17	6.90_d_	0.18	85.61_a_	2.29	0.57_b_	0.03	51.95_a_	1.38	11.98_a_
TAG	14.11_c_	0.40	16.30_a_	0.40	69.53_b_	1.98	0.85_b_	0.04	46.16_b_	1.30	4.93_b_
PL	27.13_b_	0.82	10.43_c_	0.28	62.31_c_	1.50	1.13_b_	0.03	3.10_c_	0.11	2.30_c_
SE	55.74_a_	1.85	12.06_b_	0.32	32.20_d_	0.88	24.05_a_	0.75	4.74_c_	0.14	0.58_d_
**CO9**											
TL	7.04_d_	0.14	5.63_c_	0.16	86.94_a_	2.34	0.90_b_	0.03	54.80_a_	1.38	12.35_a_
TAG	10.92_c_	0.30	10.71_a_	0.31	78.27_b_	2.15	0.63_b_	0.04	50.59_b_	1.28	7.17_b_
PL	27.52_b_	0.72	9.04_b_	0.28	63.34_c_	1.52	1.39_b_	0.02	3.12_c_	0.06	2.30_c_
SE	52.96_a_	1.60	10.97_a_	0.30	36.07_d_	1.05	24.58_a_	0.65	3.69_c_	0.07	0.68_d_
**CO10**											
TL	6.74_d_	0.16	5.64_c_	0.14	87.22_a_	2.55	0.77_b_	0.03	54.52_a_	1.35	12.95_a_
TAG	11.47_c_	0.31	10.94_a_	0.28	77.52_b_	2.20	0.58_b_	0.03	49.59_b_	1.18	6.76_b_
PL	26.32_b_	0.72	8.69_b_	0.25	64.90_c_	1.55	1.04_b_	0.02	3.04_c_	0.08	2.47_c_
SE	54.07_a_	1.65	10.34_a_	0.26	35.59_d_	0.95	24.60_a_	0.68	3.43_c_	0.08	0.66_d_
**CO11**											
TL	7.34_d_	0.18	5.48_d_	0.12	86.87_a_	2.60	0.70_b_	0.03	55.93_a_	1.40	11.83_a_
TAG	11.96_c_	0.30	10.18_b_	0.25	77.77_b_	2.25	0.55_b_	0.04	50.33_b_	1.20	6.50_b_
PL	28.45_b_	0.70	9.49_c_	0.22	62.02_c_	1.52	1.07_b_	0.03	2.47_c_	0.08	2.18_c_
SE	53.90_a_	1.75	11.07_a_	0.27	35.03_d_	0.90	24.78_a_	0.80	3.19_c_	0.09	0.65_d_

Values are given as mean *±* SD of three samples, analyzed individually in triplicate (n = 3x3).

Means in the same column followed by different subscript letters indicate significant differences (*p* < 0.05) among lipid classes of each genotype of pot marigold (ANOVA "Tukey's Multiple Comparison Test"). TL - total lipids, TAG- triacylglycerols, PL- polar lipids, SE- sterol esters. SFAs- saturated fatty acids, MUFAs- monounsaturated fatty acids, PUFAs- polyunsaturated fatty acids, VLCSFAs- very long chain saturated fatty acids, ∑CLNAs [18:3 (8*trans*, 10*trans*, 12*cis*) + 18:3 (8*trans*,10*trans*,12*trans*)] - conjugated linolenic acids.

#### TL fatty acids

**Figure 2:**
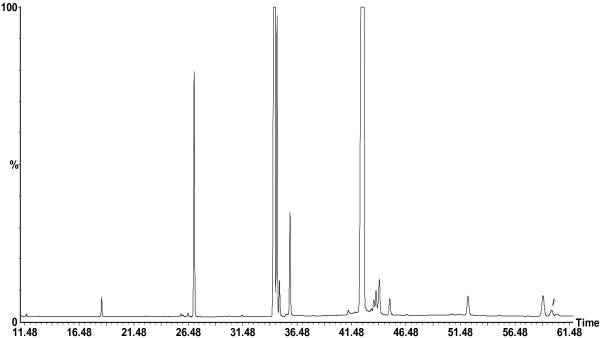
**GC-MS chromatogram of FAMEs in the TLs of *Calendula officinalis *L. (CO2: cv. Prolifera nr. 214) seeds analysed with a BPx- 70 capillary column.** Peaks: (1) lauric (12:0), (2) myristic (14:0), (3) pentadecanoic (15:0), (4) *cis*-7 hexadecenoic [16:1 (n-9)], (5) palmitoleic [16:1 (n-7)], (6) palmitic (16:0), (7) margaric (17:0), (8) linoleic [18:2 (n-6)], (9) oleic [18:1 (n-9)], (10) elaidic [18:1 (9 *t*) (n-9)], (11) linoelaidicic [18:2 (9 *t*,12 *t*) (n-6)], (12) stearic (18:0), (13) α- linolenic [18:3 (n-3)], (14) calendic [18:3 (8 *t*, 10 *t*, 12*c*) (n-6)], (15) gondoic [20:1 (n-9)], (16) β- calendic [18:3 (8 *t*, 10 *t*, 12 *t*) (n-6)], (17) arachidic (20:0), (18) 9- hydroxy- *trans*-10, *cis*-12 octadecadienic (9- HODE), (19) behenic (22:0) acids.

Nineteen fatty acids were identified in the studied pot marigold seed oils (Figure 2), including very low amounts of a hydroxy fatty acid, namely 9- hydroxy- *trans*-10, *cis*-12 octadecadienic-acid (9-HODE).

As expected, calendic acid [18:3 (8 *t*, 10 *t*, 12*c*) (n-6)] was the predominant polyunsaturated fatty acid (PUFA) in all TL extracts, and its composition varied between 51.47% (in CO8) and 57.63% of total fatty acids (in CO4). The next most abundant fatty acid was linoleic acid [18:2 (n-6)] (28.50 to 31.86%), followed by oleic [18:1 (n-9)] (4.44 to 6.25%) and palmitic acids (16:0) (3.86 to 4.55%). Small and very small (or trace) amounts (<2%) of stearic (18:0), β- calendic [18:3 (8 *t*, 10 *t*, 12 *t*) (n-6)], elaidic [18:1 (9 *t*) (n-9)], arachidic (20:0), behenic (22:0), gondoic [20:1 (n-9)], α- linolenic [18:3 (n-3)], linoelaidicic [18:2 (9 *t*,12 *t*) (n-6)], *cis*-7 hexadecenoic [16:1 (n-9)], palmitoleic [16:1 (n-7)], lauric (12:0), myristic (14:0), pentadecanoic (15:0), and margaric (17:0) acids were also determined. Similar results for the calendic acid content (over 50%) were reported by Cromack and Smith [25] for two of nine hybrids of pot marigold seeds grown in England, as well as by Cahoon et al. [26]. Ozgul- Yucel concluded that Turkish calendula seed oil is characterized by high concentration of linoleic acid (43.5%) and low content of CLNAs (calendic acid (18.3%) + β- calendic (11.2%)) [34]. Moreover, the calendic acid levels reported here are considerably higher than those reported previously by Suzuki et al. [29] (33.4%) and Angelini et al. [35] (16- 46%- in the Italian pot marigold seed oils, crops from 1993).

The available literature shows that the fatty acid composition of oil seeds varies strongly according to their origin/genotype, and geographical/climatic conditions of the growth areas [25,36]. It was also found that the maturity stage of the seeds is an important factor that influences the accumulation of calendic acid in calendula seeds oil. Pintea et al. [33] showed that during the maturation period of the pot marigold seeds (0–2 weeks after flower drops) the concentration of calendic acid increased sharply and steadily (from 8.62% to 53%), accompanied by a decrease in the amounts of linoleic and oleic acids. These observations are in agreement with the presence of the specific conjugase which is able to convert linoleic acid into calendic acid in *Calendula* seeds [18,26]. The stereospecific analysis of TAG proved that calendic acid preferentially esterifies the *sn*-2 position of TAG [26,37].

**Figure 3:**
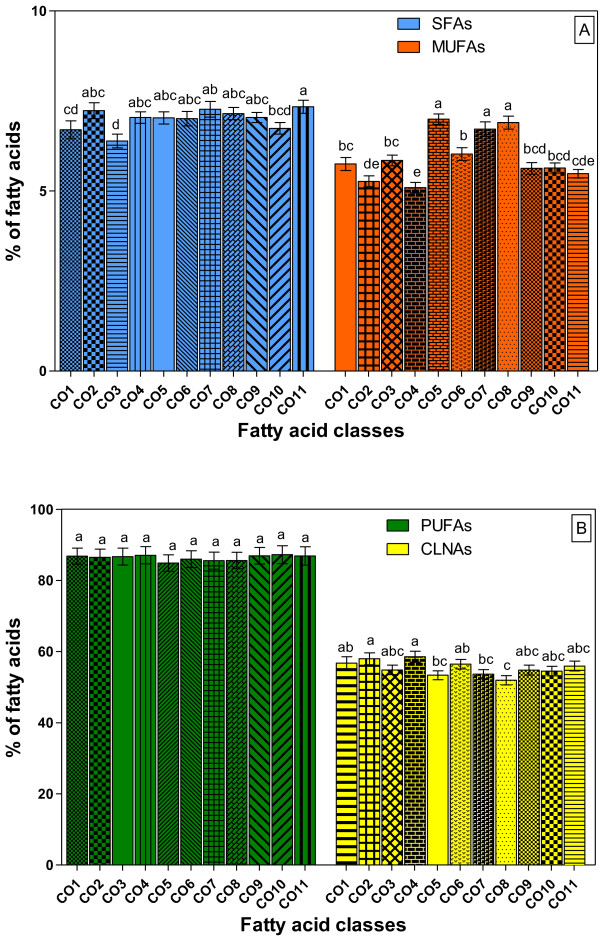
**Comparative representation of fatty acid classes from total lipids of different genotypes of pot marigold (*Calendula officinalis *L.) seed oils.** CO1- CO11, pot marigold (*Calendula officinalis* L.) genotypes. Values are mean *±* SD of three samples, analyzed individually in triplicate (n = 3x3). Values with different letters (a-e) are significantly different (*p* < 0.05), using ANOVA "Tukey's Multiple Comparison Test". SFAs- saturated fatty acids, MUFAs- monounsaturated fatty acids, PUFAs- polyunsaturated fatty acids, CLNAs- conjugated linolenic acids.

The analysis of fatty acids classes showed statistically significant differences (*p* < 0.05) with the exception of PUFAs (Figure 3). The highest value of saturated fatty acid (SFAs) (*p* < 0.05) was registered in the TLs of Czech genotypes (CO11) (7.34%), whereas CO5, CO7 and CO8 were the richest sources of monounsaturated fatty acids (MUFAs) (Figure 3A). On the other hand, small variations (*p* < 0.05) were found in CLNAs contents (Figure 3B), with the highest proportions in CO4 (58.54%) and the lowest in CO8 (51.95%), respectively. As shown in Table 2, the levels of the PUFAs/SFAs (saturated fatty acids) ratios were significantly higher (*p* < 0.05) in TLs (due to the high values of 18:3 and 18:2 fatty acids) than in the lipid fractions (TAGs, PLs and SEs) of each pot marigold genotypes.

#### TAG fatty acids

The fatty acid profiles of the TAGs were similar to that of the profiles of the TL fractions, due to the dominance of the PUFAs (18:3 and 18:2 (n-6) fatty acids) in their compositions (see Tables 1 and 2) and due to the fact that TAG are major components of the seeds oil.

#### PL and SE fatty acids

The fatty acid composition of the PLs and SEs was different from that of the TL and TAG fractions in all the pot marigold genotypes analyzed (Tables 1 and 2).

The PL fractions were highly unsaturated, with the linoleic acid content ranging from 55.02% (CO2) to 61.51% (CO10) of total fatty acids. Ul’chenko et al. [32] studied the fatty acid compositions of the lipids from seeds, leaves and flowers of *Calendula officinalis* L. and reported lower value of linoleic acid (24.5%) in the phospholipids of seeds, than those determined in the present work.

With four exceptions (samples CO1-4), the calendic acid content in the PL fractions was lower than 3% (Table 1). This conjugated fatty acid was found to be below 1% in the phosphatidylcholine (PC) of *Calendula officinalis* seeds oil [26] or was not detected [32]. The differences between the reported data and our data can be explained by the fact that we have investigated the total polar lipids fraction which includes phospholipids and glycolipids. Transgenic soybean and *Arabidopsis* seeds engineered to synthesize calendic acid (by cloning of the fatty acid conjugase from *Calendula*) accumulated moderate level of conjugated fatty acids. Calendic acid was found at comparable levels in PC and TAG fractions (85% in the *sn-*2 position of PC) proving that complex mechanisms involving both desaturation and transacylation processes are involved in the biosynthesis of rich CLNAs enriched TAG [26]. Same authors showed that accumulation of conjugated fatty acids in PC of transgenic plants (soybean and *Arabidopsis*) negatively affected the appearance and the germination rate of seeds due to the special chemical and physical properties of CLNAs. In consequence, the selection of valuable genotypes of *Calendula* which are able to produce large amounts of oil enriched in CLNAs still has an economical importance.

The levels of SFAs in SEs were significantly higher (p *<* 0.05) than in the corresponding lipid fractions of each genotype (Table 2). The amounts of saturated consisted mainly of palmitic (16:0) acid, very long-chain saturated fatty acids (VLCSFAs) (more than 20 carbon atoms) and stearic (18:0) acid, respectively, and varied between 49.31% (in CO1) and 55.74% (in CO8) of total fatty acids from SEs (Tables 1 and 2). These observations are in agreement with the data reported by Zlatanov [38], Kallio et al. [39] and Yang et al. [40] about the fatty acid composition of the phospholipids and the SE fractions of other non-conventional seed oils.

In plant tissues, the very long-chain fatty acids (≥20 carbon atoms) are precursors for the synthesis of lipids, such as cuticular waxes (on the aerial plant surfaces), suberin (embedded in the cell walls of plant-environment interfaces), triacylglycerols (in seeds), and ceramides (in the cell membranes) [41,42].

The TL, TAG and PL fractions of all analyzed pot marigold genotypes exhibited very low proportions of VLCSFAs (<1.50% of total fatty acids), whereas the SEs showed significantly higher (p *<* 0.05) amounts of this type of fatty acids (from 20.59% (CO4) to 24.78% (CO11)) (Table 2).

As shown in Table 2, in all extracts of pot marigold seeds, the PUFAs/SFAs ratios were significantly lower (p *<* 0.05) in SE and PL fractions than in the corresponding TLs or TAGs. A comprehensive study of the Diabetes and Nutrition Study Group of the Spanish Diabetes Association showed that a dietary PUFAs/SFAs ratio > 0.4 can greatly reduce the risk of onset of diabetic complications [43]. Moreover, in some earlier reports, the authors indicate that the values of this ratio comprised between 1.0 and 1.5, are optimal to reduce the risk of cardiovascular diseases [44,45]. Thus, the results of the present study show that the *Calendula officinalis* oil, whatever the genotype analyzed in this paper, may reduce the risk of cardiovascular diseases because both TLs and TAG presented PUFAs/SFAs ratios values are closed to the recommended PUFA/SFA intake by nutrition scientists.

## Conclusions

In the present paper, seeds of eleven genotypes of *Calendula officinalis* L. originating from six different locations in Europe, cultivated in Romania (Transylvanian) were analyzed with respect to oil yields and fatty acid contents. To the best of our knowledge, data about detailed fatty acid composition of main lipid fractions in pot marigold seeds investigated in this study are not available in literature.

The oil content observed in most of the calendula seed samples studied was noted to range between 18 and 22 g oil/100 g seeds. The oil TAGs were similar in fatty acid composition to the TLs, containing substantial amounts of calendic and linoleic acids, making them excellent dietary sources of PUFAs, especially of CLNAs. The PL fractions were highly unsaturated, due to the dominance of the linoleic acid in their structures. A clear characteristic of the SEs from the pot marigold seed oils analyzed were the significantly high levels of SFAs, with considerable amounts of VLCSFAs.

The compositional differences between the genotypes should be considered when breeding and exploiting the calendula seeds for industrial, nutraceutical or pharmacological purposes.

## Materials and methods

### Seeds and chemicals

Eleven genotypes of *Calendula officinalis* L. originating from six different locations in Europe (botanical gardens and institutes) (Table 3) were cultivated on experimental fields of the University of Agricultural Sciences and Veterinary Medicine of Cluj- Napoca (Romania). The crops were established in the first half of May 2011, to a target population of 40 plants m^-2^. Plot area was prepared before (autumn of 2010) by fertilization with animal manure. Nitrogen- based fertilizers were applied during the vegetation period. The seeds were harvested manually at full maturity (end of September-beginning of October).

**Table 3:** **Genotypes of *Calendula officinalis *L. (CO) evaluated**

**Samples**	**Genotypes**	**Sources**
CO1	*C. officinalis* L. D.a	Humboldt-Universität zu Berlin, Institut für Biologie, Germany
CO2	cv. Prolifera nr. 214	Botanische Garten der Universität Göttingen, Germany
CO3	Bon-Bon Orange	National Botanic Garden of Latvia, Salaspils, Latvia
CO4	Bon-Bon Mix’	Hortus Botanicus Fominianus, Kiev, Ukraine
CO5	cv. Radio	Ökologisch-Botanischer Garten der Universität Bayreuth, Germany
CO6	*C. officinalis* L. PL	Hortus Farmacognosticus Academiae Medicinalis, Lublin, Poland
CO7	*C. officinalis* L. I	Instituto di Botanica e Orto Botanico Pierino Scaramella, Italy
CO8	cv. Prycosnovjenie	National Botanical Gardens Timirjazevska, Kiev, Ukraine
CO9	cv. Pacific Beauty	National Botanical Gardens Timirjazevska, Kiev, Ukraine
CO10	cv. Zelenoye Serdtse	National Botanical Gardens Timirjazevska, Kiev, Ukraine
CO11	cv. Plamen	Masarykova Univerzita Brno, Czech Republic

All reagents (used for the oil extraction, fractionation and fatty acid methyl esters (FAMEs) preparation) and lipid standards (used for identification of the lipid class) were of chromatographic grade (Sigma–Aldrich (St. Louis, MO, USA)). The thin layer chromatography (TLC) plates (silica gel 60 F254, 20 × 20 cm) were purchased from Merck (Darmstadt, Germany).The FAMEs standard (37 component FAME Mix, SUPELCO, catalog No: 47885-U) were purchased from Supelco (Bellefonte, PA, USA).

### Oil extraction and fractionation

The oils were extracted from 5 g of seeds, using a methanol/chloroform extraction procedure, according to Yang et al. [36] and Dulf et al. [46]. The sample was homogenized in 50 mL methanol for 1 min using a homogeniser (MICCRA D-9, Germany), 100 mL chloroform was added, and homogenization was continued for further 2 min. The mixture was filtered under vacuum through a Buchner funnel and the solid residue was resuspended in 150 mL of chloroform: methanol (2:1, v/v) and homogenized for another 3 min. The mixture was filtered again and washed with 150 mL chloroform: methanol (2:1, v/v). The filtrates were combined and cleaned with 0.88% potassium chloride water solution and methanol: water (1:1, v/v) solution. The bottom layer (with the purified lipids) was filtered before the solvent was rotary evaporated. The total lipids recovered were transferred to vials with 4 mL chloroform (stock solution), and stored at −18°C for further analysis.

Neutral and polar lipid fractions were separated by TLC [47]. Lipid aliquots (0.2 ml of stock solution) were applied on the TLC plates and then developed in a mixture of petroleum ether: diethyl ether: acetic acid (85:15:1, v/v/v), sprayed with 2’, 7’-dichlorofluoroscein/methanol (0.1% w/v) and viewed under UV light (254 nm) [48]. The lipid classes were identified using commercial standards and then scraped from the TLC plates. The first band (at the origin of the plates), corresponding to the PLs was eluted from silica layer with methanol: chloroform (1:1, v/v), and the upper two major bands of TAGs and SEs respectively were eluted with chloroform. The samples were filtered, the solvent was removed and the dry residue was subjected to transesterification and gas chromatographic (GC) analysis.

### Fatty acid analysis

The total lipid, PL, TAG and SE fractions were derivatized by sodium methoxide catalysis [49]. The FAMEs were determined by gas chromatography–mass spectrometry (GC-MS), using a PerkinElmer Clarus 600 T GC-MS (PerkinElmer, Inc., Shelton, U.S.A.) equipped with a, BPx- 70 capillary column (60 m *×* 0.25 mm i.d., 0.25 μm film; SGE, Ringwood, Australia). The initial oven temperature was 140°C, increased to 220°C with a rate of 2°C/min and then held at this temperature for 25 min. Flow rate of the carrier gas He and the split ratio were 0.8 ml/min and 1:24, respectively. The injector temperature was 210°C. The positive ion electron impact (EI) mass spectra was recorded at an ionization energy of 70 eV and a trap current of 100 μA with a source temperature of 150°C. The mass scans were performed within the range of m/z: 22–395 at a rate of 0.14 scan/s with an intermediate time of 0.02 s between the scans. The injected volume was 0.5 μl. Identification of FAMEs was achieved by comparing their retention times with those of known standards (37component FAME Mix, SUPELCO # 47885-U) and the resulting mass spectra to those in our database (NIST MS Search 2.0).

### Statistics

Three different samples of *Calendula* seeds for each genotype were assayed. The analytical results reported for the fatty acid compositions, are the average of triplicate measurements of three independent oils (n = 3x3). The assumptions of equality of variances and normal distribution of errors were checked for the tested response variables. Since the assumptions were satisfied, data were subjected to one-way ANOVA (repeated measures ANOVA) and Tukey’s post hoc test. Statistical differences among oil samples were estimated using: “Kruskal-Wallis non-parametric test” followed by “Dunn's Multiple Comparison Test” (Graph Pad Prism Version 4.0, Graph Pad Software Inc., San Diego CA). A probability value of *p* < 0.05 was considered to be statistical significant.

## Abbreviations

CLNAs: Conjugated linolenic acids; TLs: Total lipids; TAGs: Triacylglycerols; PLs: Polar lipids; SEs: Sterol esters; PUFAs: Polyunsaturated fatty acids; SFAs: Saturated fatty acids; MUFAs: Monounsaturated fatty acids; VLCSFAs: Very long-chain saturated fatty acids; PC: Phosphatidylcholine; CO: *Calendula officinalis*; FAMEs: Fatty acid methyl esters; TLC: Thin layer chromatography; GC-MS: Gas chromatography–mass spectrometry.

## Competing interests

The authors declare that they have no competing interests.

## Authors’ contributions

FVD and DP carried out the experimental design, interpretation of results and preparation of the paper. ADB contributed to the extraction of lipids. AP contributed to the separation, identification and quantification of the lipid fractions and fatty acids from the samples. All authors read and approved the final manuscript.
